# Ischemic stroke with unknown onset of symptoms: current scenario and perspectives for the future

**DOI:** 10.1055/s-0042-1755342

**Published:** 2022-12-29

**Authors:** Rônney Pinto Lopes, Vivian Dias Baptista Gagliardi, Felipe Torres Pacheco, Rubens José Gagliardi

**Affiliations:** 1 Irmandade da Santa Casa de Misericórdia de São Paulo, São Paulo, Brazil.; 2 Universidade Federal de São Paulo, Departamento de Neurologia e Neurocirurgia, São Paulo SP, Brazil.; 3 Diagnósticos da América SA, Departamento de Imagem Médica, Divisão de Neurorradiologia, São Paulo SP, Brazil.; 4 Santa Casa de São Paulo, Faculdade de Ciências Médicas, Divisão de Neurologia, São Paulo SP, Brazil.

**Keywords:** Ischemic Stroke, Neuroimaging, Tissue Plasminogen Activator, Thrombectomy, AVC Isquêmico, Neuroimagem, Ativador de Plasminogênio Tecidual, Trombectomia

## Abstract

**Background**  Stroke is a major cause of disability worldwide and a neurological emergency. Intravenous thrombolysis and mechanical thrombectomy are effective in the reperfusion of the parenchyma in distress, but the impossibility to determine the exact time of onset was an important cause of exclusion from treatment until a few years ago.

**Objectives**  To review the clinical and radiological profile of patients with unknown-onset stroke, the imaging methods to guide the reperfusion treatment, and suggest a protocol for the therapeutic approach.

**Methods**  The different imaging methods were grouped according to current evidence-based treatments.

**Results**  Most studies found no difference between the clinical and imaging characteristics of patients with wake-up stroke and known-onset stroke, suggesting that the ictus, in the first group, occurs just prior to awakening. Regarding the treatment of patients with unknown-onset stroke, four main phase-three trials stand out: WAKE-UP and EXTEND for intravenous thrombolysis, and DAWN and DEFUSE-3 for mechanical thrombectomy. The length of the therapeutic window is based on the diffusion weighted imaging–fluid-attenuated inversion recovery (DWI-FLAIR) mismatch, core-penumbra mismatch, and clinical core mismatch paradigms. The challenges to approach unknown-onset stroke involve extending the length of the time window, the reproducibility of real-world imaging modalities, and the discovery of new methods and therapies for this condition.

**Conclusion**  The advance in the possibilities for the treatment of ischemic stroke, while guided by imaging concepts, has become evident. New studies in this field are essential and needed to structure the health care services for this new scenario.

## Resumo

**Antecedentes**  O acidente vascular cerebral (AVC) é uma das principais causas de incapacidade em todo o mundo, e uma emergência neurológica. A trombólise intravenosa e a trombectomia mecânica são eficazes na reperfusão do parênquima em sofrimento, mas a impossibilidade de determinar o tempo exato de início era uma causa importante de exclusão ao tratamento até alguns anos atrás.

**Objetivos**  Revisar o perfil clínico-radiológico dos pacientes com AVC de tempo indeterminado, os métodos de imagem para guiar o tratamento de reperfusão, e sugerir um protocolo para a abordagem terapêutica.

**Métodos**  Os diferentes métodos de imagem foram agrupados de acordo com os tratamentos atuais baseados em evidências.

**Resultados**  A maioria dos estudos não encontrou diferença entre as características clínicas e de imagem dos pacientes com AVC reconhecido ao despertar e AVC de tempo definido, o que sugere que o icto, no primeiro grupo, ocorre próximo ao acordar. Quanto ao tratamento do AVC de tempo indeterminado, quatro grandes estudos na fase três sobressaem: WAKE-UP e EXTEND para trombólise intravenosa, e DAWN e DEFUSE-3 para trombectomia mecânica. A ampliação da janela terapêutica fundamenta-se nos paradigmas de incompatibilidade da imagem ponderada de difusão–recuperação da inversão atenuada por fluidos ( *diffusion weighted imaging–fluid-attenuated inversion recovery* , DWI-FLAIR, em inglês), do núcleo isquêmico e penumbra, e clínico-radiológico. Os desafios na abordagem do AVC de tempo indeterminado envolvem a ampliação da janela terapêutica, a reprodutibilidade das modalidades de imagem no mundo real, e a identificação de novos métodos e tratamentos para essa condição.

**Conclusão**  É evidente o avanço nas possibilidades de tratamento do AVC isquêmico guiado pelos conceitos de imagem. Novos estudos nesse campo são essenciais, com necessidade de estruturar os serviços de saúde para esse novo cenário.

## INTRODUCTION

 Stroke is one of the main causes of disability worldwide, and it consists of an episode of acute neurological dysfunction presumably due to ischemia or bleeding persisting for more than 24 hours or leading to death. 1

 Due to the social and economic impacts of strike, studies published in recent decades have tried to exhaustively assess the benefit of treatments that could change the progression of the disease and, thus, reduce the permanent sequelae in individuals. Regarding that, intravenous thrombolysis 2 3 4 and mechanical thrombectomy 5 6 7 8 9 proved to be effective in the reperfusion of the brain parenchyma suffering from ischemic distress. 

 However, both treatments are underused worldwide, mainly due to the patient's late admission to emergency services, as per the time window parameter according to which the therapies were approved in agreement with national 10 and international guidelines. 11 12 Among the factors leading to this problem, the impossibility of determining the moment of onset of symptoms that can be attributed to stroke by either the patient or a witness stands out in some cases. 

 One in five stroke patients wakes up with neurological deficits and, because of that, they are unable to specify the onset of the ictus. 13 In addition, another portion of patients presents with symptoms that make it impossible for them to communicate with the medical team, which in turn deprives them from receiving the most suitable treatment. 

From the perspective of this complex issue, new studies have sought to perform in-depth analyses of the characteristics of this subpopulation and find objective ways to safely extend the therapeutic window in ischemic stroke – and thus benefit more individuals. We will herein review the clinical and radiological profile of patients with unknown-onset stroke, the imaging methods used to guide the reperfusion treatment, and suggest a therapeutic approach protocol. The different methods will be grouped according to the treatment possibilities currently offered in acute ischemic stroke: intravenous thrombolysis and mechanical thrombectomy.

## CLINICAL AND EPIDEMIOLOGICAL CHARACTERISTICS OF UNKNOWN-ONSET STROKE

Unknown-onset stroke includes all conditions in which an exact time of onset of neurological symptoms cannot be established. More specifically, the patient may have gone to sleep well and woke up presenting with symptoms of stroke or is unable to communicate the time of onset of ictus due to aphasia or a change in their level of consciousness, and there is no available witness. For the purpose of understanding this review, the first group will be referred to as a wake-up stroke and the second, as a daytime-unwitnessed stroke.

 The study by Dekker et al. 14 showed no difference in the functional outcome and radiological evaluation (using the Alberta Stroke Program Early CT Score, ASPECTS, and a collateral system) between patients with wake-up stroke and patients with daytime-unwitnessed stroke. It should be noted that it took the first subgroup 2.5 hours longer than the second subgroup to present to the emergency service. 

 Other previous studiesn 15 16 17 18 did not show significant differences regarding demographic characteristics, vascular risk factors, and the clinical severity of patients presenting with wake-up stroke or known-onset stroke. However, a population-based study 19 found that patients who woke up presenting stroke-related symptoms were older and had a more severe condition as compared with patients who were awake at the onset of symptoms. 

 A study 15 based on non-contrast computed tomography (NCCT) assessment showed no difference between hyperacute ischemic changes in patients who woke up with stroke symptoms within three hours of the awareness thereof and patients with known-onset stroke. 

 Another study, 20 with results similar to those of the aforementioned one, 15 made an additional comparison of patients with stroke of known onset and at awakening with a third group of patients with daytime-unwitnessed stroke, revealing that this last group had better defined hypodense areas than did the first two groups. These radiological characteristics are in accordance with those from the study by Reid et al., 17 which showed worse clinical severity and outcome in patients with daytime-unwitnessed stroke, rather than wake-up stroke, in relation to patients with known-onset stroke. 

 Dankbaar et al. 21 compared wake-up stroke patients who were last seen well > 4.5 hours and ≤ 4.5 hours to patients with a known time of symptom onset ≤ 4.5 hours. Although the ASPECTS score was lower in the > 4.5h wake-up stroke patients as compared with patients with a known onset time, 75% of patients with wake-up stroke had favorable ASPECTS scores and good filling of the leptomeningeal collaterals on CT angiography (CTA). 

 There was also an additional analysis among patients who woke up presenting neurological deficits and proximal occlusion of the anterior circulation last seen well for > 6 hours and for ≤ 6 hours, who were compared with patients with known-onset stroke within ≤ 6h and proximal occlusion. 21 In this context, 57% of wake-up stroke patients with proximal occlusions last seen well for > 6h had an ASPECTS score higher than 7 and good collateral filling. 

Taken together, these studies suggest that wake-up stroke actually occurs during the early hours of the morning, moments before the patient or any witnesses can recognize the symptoms. This concept has direct implications for the treatment of this subgroup of patients, as it would make them possibly eligible for reperfusion therapies.

 It is noteworthy that, although there is agreement regarding the results in most studies, there are some limitations thereto. Some of these results come from single-center 14 15 16 17 and retrospective studies. 18 19 20 Moreover, the inclusion and exclusion criteria varied among them. 

## ADVANCED NEUROIMAGING IN UNKNOWN-ONSET STROKE

 The main goal when approaching the acute phase of stroke is to restore cerebral blood flow as soon as possible. Bearing that in mind, the hallmark in treating this condition occurred in 1995, when a trial by the National Institute of Neurological Disorders and Stroke (NINDS) 22 showed the benefit of using recombinant tissue plasminogen activator (rt-PA) in patients within 3 hours of symptom onset. 

 Subsequent to the NINDS trial, the European Cooperative Acute Stroke Study (ECASS), 23 ECASS II, 24 Alteplase Thrombolysis for Acute Noninterventional Therapy in Ischemic Stroke (ATLANTIS A), 25 and ATLANTIS B 26 studies included stroke patients within a time period of 3 hours to 6 hours, but were individually negative as to the prespecified primary outcome. Only in 2008 did the ECASS III study 27 find a better functional outcome in the group treated with rt-PA within the window of 3 hours to 4.5 hours when compared with placebo, despite emphasizing that the thrombolytic treatment is time-dependent and shows better outcome in patients treated earlier. 

 Endovascular thrombectomy (EVT), in turn, proved to be effective in patients with stroke due to large vessel occlusion (LVO), notably in 2015, with the publication of five important clinical trials: Endovascular Treatment for Small Core and Anterior Circulation Proximal Occlusion With Emphasis on Minimizing CT to Recanalization Times (ESCAPE), 5 Solitaire FR with the Intention for Thrombectomy as Primary Endovascular Treatment for Acute Ischemic Stroke (SWIFT PRIME), 6 Extending the Time for Thrombolysis in Emergency Neurological Deficits - Intra-Arterial (EXTEND-IA), 7 Multicenter Randomized Clinical Trial of Endovascular Treatment for Acute Ischemic Stroke in the Netherlands (MR CLEAN), 8 and Endovascular Revascularization with Solitaire Device versus Best Medical Therapy in Anterior Circulation Stroke within 8 Hours (REVASCAT). 9 A meta-analysis of these studies, derived from the Highly Effective Reperfusion Using Multiple Endovascular Devices (HERMES) collaboration, showed a significant reduction in disability at 90 days in the EVT group compared with the controls (adjusted common odds ratio [cOR]: 2.49; 95% confidence interval [95%CI]: 1.76–3.53; *p*  < 0.0001). The number needed to treat (NNT) for one additional patient to achieve a 1-point reduction in the modified Rankin scale (mRS) was 2.6. 28

 However, until the 2013 American Heart Association/American Stroke Association (AHA/ASA) guidelines, 29 intravenous rt-PA and mechanical thrombectomy were recommended for patients within 4.5 hours (class I; level of evidence B) and 6 hours (class I; level of evidence B) of symptom onset respectively, excluding patients with unknown-onset stroke. 

To also extend the treatment to this subgroup of patients, whose time of stroke-related symptoms onset cannot be determined, most studies over recent decades have focused on the assessment of advanced neuroimaging methods regarding the identification of patients who might benefit from reperfusion therapies.

For the sake of clarity, the main evidence regarding the reperfusion treatment based on advanced imaging paradigms will be subdivided relative to the therapy studied.

## INTRAVENOUS THROMBOLYSIS IN UNKNOWNONSET STROKE

The main studies related to the use of intravenous thrombolysis in patients with unknown-onset stroke were based on two imaging concepts: the diffusion-weighted imaging–fluid-attenuated inversion recovery (DWI-FLAIR) mismatch and the core-penumbra mismatch.

**Figure 1:**
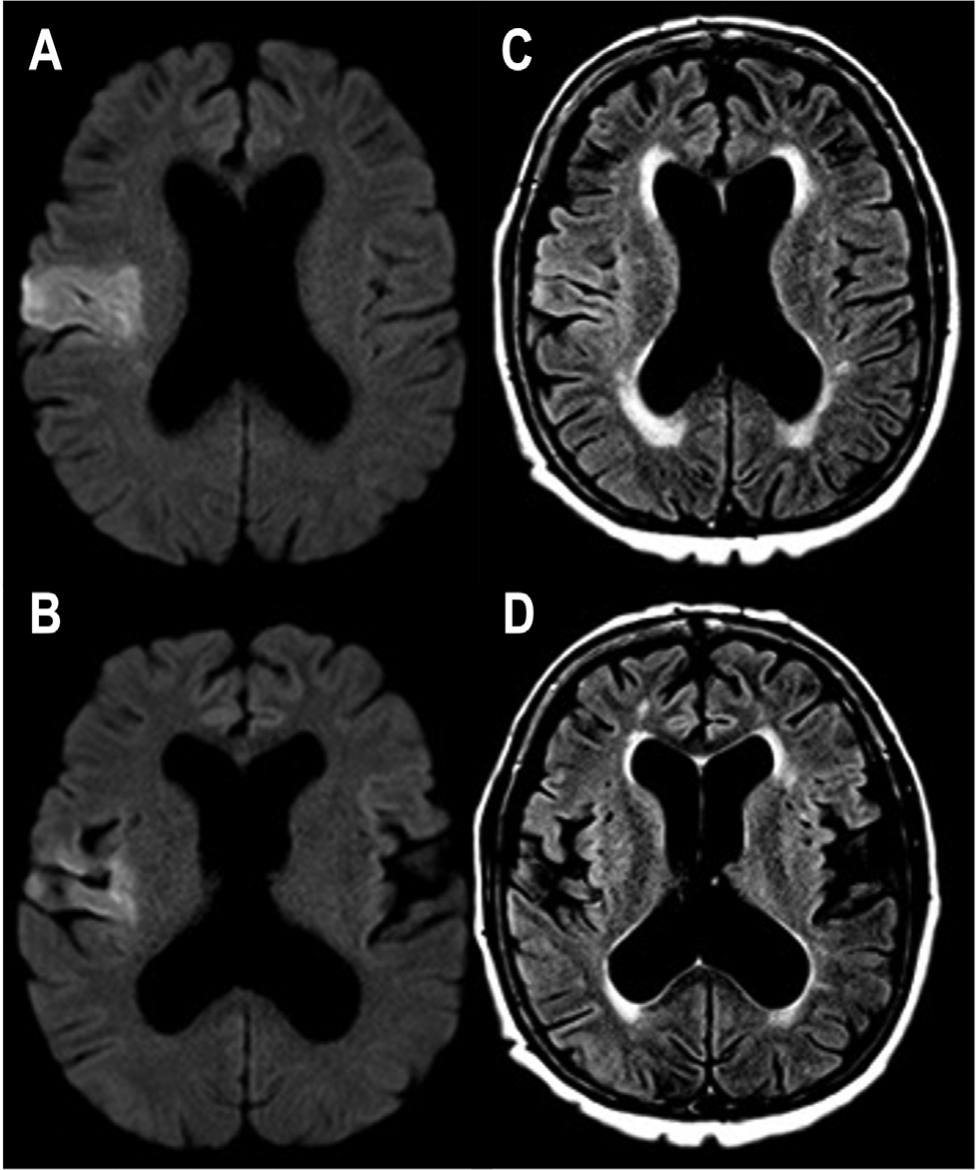
Axial diffusion-weighted imaging ( **A,B** ) demonstrating restriction in the free movement of water in the right frontoparietal region, without correspondences in FLAIR sequences at the same level ( **C,D** ), suggesting an ischemic event < 4.5 hours.

**Figure 2:**
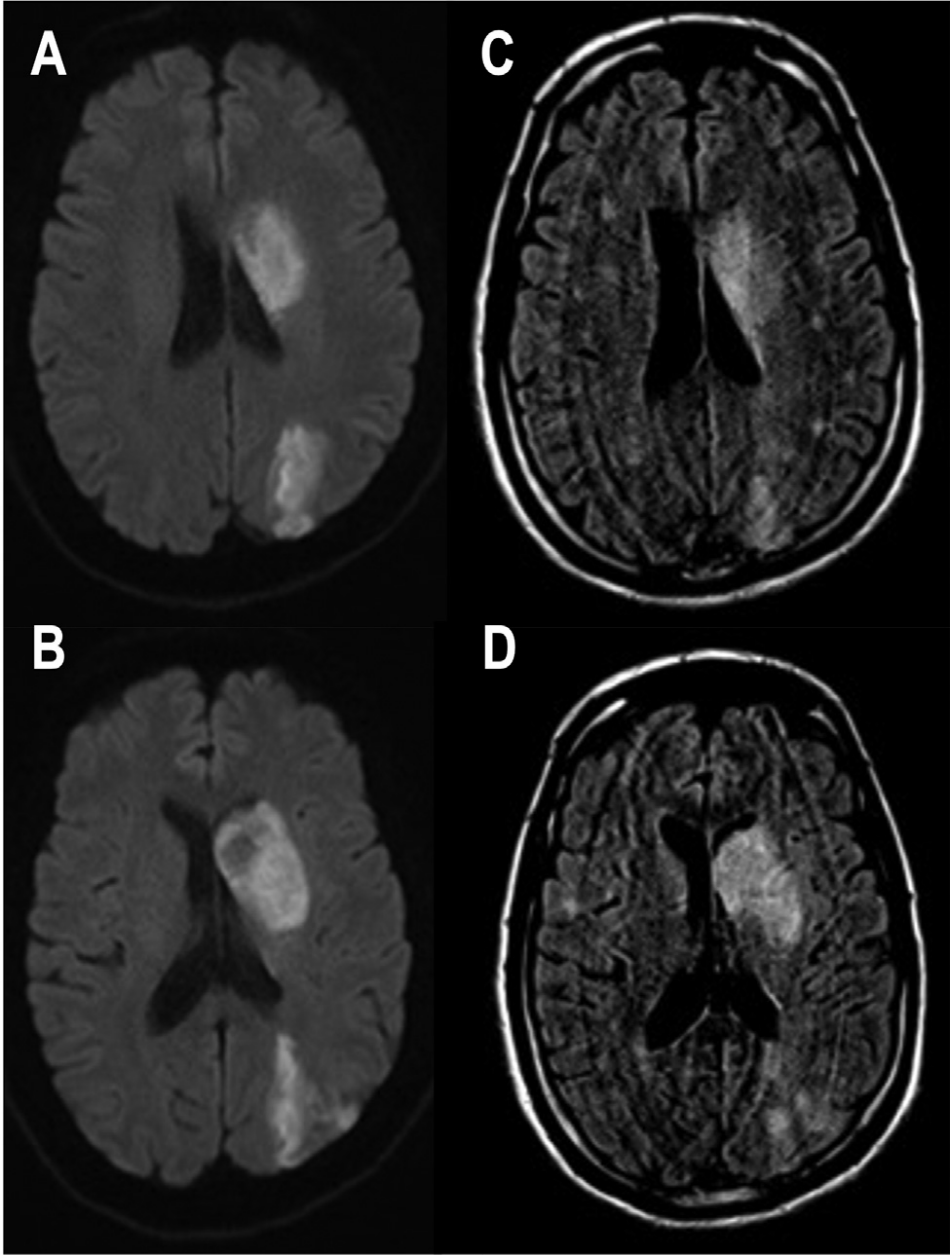
Axial diffusion-weighted imaging ( **A,B** ) demonstrating restriction in the free movement of water in the left nucleocapsular region and ipsilateral precuneus, and hypersignal in the FLAIR sequence at the same level ( **C,D** ), suggesting an ischemic event > 4.5 hours.

 The DWI-FLAIR mismatch tries to correlate signal changes in different sequences on magnetic resonance imaging (MRI) with the pathophysiological cascade of acute ischemia, in an attempt to estimate the elapsed time since the ischemic ictus. The reduction in cerebral blood flow caused by the occlusion of an intracranial artery leads to a disruption in the energy balance, failure of the sodium–potassium (Na ^+^ /K ^+^ ) pumps, and translocation of water to the intracellular space, culminating in cytotoxic edema and restriction in the movement of water molecules (hyperintensity in DWI) within the first minutes after the ischemic event. During the following hours, with the progression of ischemia, there is a blood-brain barrier disruption and shift of macromolecules into the extravascular compartments, culminating in FLAIR hyperintensity. Thus, the presence of a lesion in DWI (positive) and absence of a corresponding altered area in FLAIR (negative) estimates an ischemia onset time at < 4.5 hours ( Figures 1 and 2 ). 30

 The Identification of Stroke Patients ≤ 3 and ≤ 4.5 Hours of Symptom Onset by FLAIR Imaging and DWI (PRE-FLAIR) study 31 showed that the DWI-FLAIR mismatch could be used to identify stroke patients within 4.5 hours with a sensitivity of 62%, specificity of 78%, positive predictive value of 83%, and negative predictive value of 54%. The main studies that tried to distinctly assess this concept were the Study of Intravenous Thrombolysis with Alteplase in MRI-Selected Patients (MR WITNESS), 32 Efficacy and Safety of MRI-based Thrombolysis in Wake-up Stroke (WAKE-UP), 33 and Thrombolysis for Acute Wake-up and Unclear-onset Strokes with Alteplase at 0.6 mg/kg Trial (THAWS). 34

 The MR WITNESS open trial 32 tested the safety of intravenous thrombolysis in patients who had a stroke onset time between 4.5 hours and 24 hours from the last time they were seen well, who received treatment within 4.5 hours of the recognition of symptoms. To achieve these goals, the researchers quantified the hyperintensity in the FLAIR sequence using the signal intensity ratio, obtained by dividing the region of interest (ROI) of the hyperintensity area by the ROI of the corresponding contralateral tissue with a normal appearance. Values of the signal intensity ratio lower than 1.15 were considered for inclusion in the study, that is, those showing up to 15% of relative increase in signal intensity as compared with the opposite hemisphere. Among 80 treated patients, there was only 1 (1.3%) case of symptomatic intracranial hemorrhage (sICH) and 3 (3.8%) cases of symptomatic edema, which demonstrates the safety of alteplase in selected patients with a quantitative DWI-FLAIR mismatch. 

 On the WAKE-UP trial, 33 the researchers randomized 503 patients with stroke symptoms upon awakening or with unknown onset within > 4.5 hours since the last time they were seen well, but who could be treated with alteplase if the time until symptom recognition was shorter than 4.5 hours. The eligibility criteria included evidence of an abnormal signal in the DWI sequence in association with a negative FLAIR as detected by visual inspection. Patients were excluded if they had a National Institutes of Health Stroke Scale (NIHSS) score greater than 25 and in whom mechanical thrombectomy was planned. Also excluded patients if MRI showed a lesion larger than one third of the territory of the middle cerebral artery and in whom a contraindication to thrombolysis was recognized (except for the unknown onset of symptoms). 

 The study was terminated early due to lack of funding, but it was sufficient to show that 53.3% of patients in the alteplase group and 41.8% of those in the placebo group ( *p* = 0.02) had an mRS scores ranging from 0 to 1 at 90 days. It is noteworthy that the median NIHSS score was 6 in both groups, and the median volume of the ischemic core in the DWI sequence seen in the intervention group was of only 2.0 mL. 

 The Japanese THAWS trial 34 used a neuroimaging concept similar to that of the WAKE-UP trial, 33 and evaluated the benefit of alteplase at a lower dose (0.6 mg/kg). The study was prematurely terminated with 131 of the 300 patients initially expected due to the publication of the positive results of the WAKE-UP trial. 33 The median NIHSS score was 7 in both groups. The study 34 showed no benefit from intravenous thrombolysis, with mRS scores ranging from 0 to 1 among 47.1% of the alteplase group and 48.3% of the placebo group ( *p*  = 0.892). It is noteworthy that an early discontinuation of the study, the absence of blinding, and the lower dose of medication may all have influenced the results. 

 To assess whether the quantitative method to determine the intensity in the FLAIR sequence could change the effect of thrombolytic treatment, a post hoc analysis 35 of the WAKE-UP trial 33 concluded that, in patients selected by visual assessment, those with higher signal intensity ratio had worse clinical outcomes. However, this result should be interpreted with caution, as it was not the aim of the study 35 to assess differences in treatment among subgroups, but rather to primarily use a paradigm that would more quickly exclude patients with FLAIR hyperintensity and who would likely not benefit from thrombolysis. 

 Guided by the results of the WAKE-UP study 33 , the 2019 AHA/ASA guidelines 36 (class IIa; level of evidence B) recommend the use of MRI to select patients with unknown-onset stroke who might benefit from the use of alteplase. The 2021 guidelines of the European Stroke Organization (ESO; high quality of evidence, strong recommendation level) 37 also recommend that intravenous thrombolysis be performed in patients with ischemic stroke of unknown-onset, provided that they have a DWI-FLAIR mismatch and are not eligible for thrombectomy . 

In turn, the concept of core-penumbra mismatch involves the identification of an area of the brain that is at risk of progressing to ischemia, but can still be saved if the regional blood flow is promptly reestablished. Such tissue suffering distress, albeit viable, is called penumbra. The mismatch comprehends the relationship between the penumbra region and the infarcted tissue that cannot be recovered, known as the ischemic core.

 Regarding the imaging methods to assess cerebral perfusion, studies define the ischemic core differently: when using CT, the area under severely reduced cerebral blood flow is estimated – cerebral blood flow (CBF) of less than 30% in comparison to normal tissue –, whereas the MRI detects the region with increased intensity in the DWI sequence. To determine the penumbra in either method, a contrast agent is injected and, with the help of maps created with different sections of brain tissue, the region can be observed with a delay (Time-to-Maximum or Tmax) of the residual tissue function longer than 6 seconds. 38

 Based on either CT perfusion or MRI perfusion, the Echoplanar Imaging Thrombolysis Evaluation Trial (EPITHET) 39 and ECASS-4, 40 with the use of alteplase, and the Desmoteplase in Acute Ischemic Stroke (DIAS) 41 and DIAS-2 42 trials, with desmoteplase, were not successful in the attempt to extend the therapeutic window to 6 hours (EPITHET) or 9 hours (DIAS, DIAS-2, and ECASS-4), and all of them were conducted in patients with known-onset stroke, except for the ECASS-4, which also admitted patients who woke up with neurological deficits. 

 In the Extending the Time for Thrombolysis in Emergency Neurological Deficits (EXTEND) trial, 43 the researchers randomized 225 stroke patients who were to receive alteplase or placebo and were within 4.5 to 9 hours of symptom onset, or within 9 hours of the midpoint of sleep (that is, the time between sleep onset and waking up with symptoms), in the case a case of wake-up stroke was involved. The core-penumbra mismatch was defined cumulatively by the ratio between the hypoperfusion volume and an ischemic core greater than 1.2, an absolute volume difference greater than 10 mL, and an ischemic core volume lower than 70 mL. The median NIHSS score was 12 in the rt-PA group and 10 in the control group. A favorable functional outcome was achieved by 35.4% of the patients in the group treated with alteplase and in 29.5% of patients in the placebo group ( *p*  = 0.04), confirming that this is the only positive study indicating thrombolysis based on core-penumbra mismatch. 

 It is emphasized that the EXTEND 43 was terminated early after the publication of the results of the WAKE-UP trial, 33 which may reflect on its results. Also, 70% of the patients included had LVO, which would qualify them to undergo a mechanical thrombectomy according to the Endovascular Therapy Following Imaging Evaluation for Ischemic Stroke 3 (DEFUSE-3) 48 and the Diffusion Weighted Imaging or Computerized Tomography Perfusion Assessment with Clinical Mismatch in the Triage of Wake Up and Late Presenting Strokes Undergoing Neurointervention (DAWN) 47 trials, which are discussed later. In addition, the evaluation of the core-penumbra mismatch is especially indicated in cases of wake-up stroke, considering that 65% of the EXTEND 43 trial population was part of this subgroup. The previous statement, however, does not preclude the inclusion of patients with daytime-unwitnessed stroke eligible for thrombolysis as indicated by this imaging pattern. 

 The positive results from using alteplase in patients with unknown-onset stroke were confirmed by a recent meta-analysis, 44 which included 843 patients from the WAKE-UP, THAWS, EXTEND, and ECASS-4 trials, 429 (51%) of whom underwent treatment with rt-PA and, of these, 385 (90%) had wake-up stroke. The MRI was used for randomization in 714 (85%) patients. In this meta-analysis, 44 47% of the alteplase group and 39% of the control group had a favorable functional outcome ( *p*  = 0.011), with an NNT of 12. 

## MECHANICAL THROMBECTOMY IN UNKNOWNONSET STROKE

 Studies involving mechanical thrombectomy for patients with unknown-onset stroke sought to extend the 6-hour therapeutic window established in the 2015 AHA/ASA guidelines. 45 To that end, the two main studies used the core-penumbra mismatch concept, the rationale of which was already mentioned, and the clinical core mismatch. 

 The clinical core mismatch is based on the fact that the symptoms attributed to stroke are correlated with the brain tissue that is under hypoperfusion, including the ischemic core and penumbra. Therefore, large clinical deficits in areas not completely correlated with the ischemic core predict that there is an area of penumbra to be saved (positive mismatch). The pioneering study conducted by Dávalos et al. 46 concluded that an NIHSS score ≥ 8 and a DWI lesion ≤ 25 mL suggested a greater chance of infarct growth and early neurological deterioration. 

 Based on this concept, the DAWN trial 47 allocated patients with intracranial internal carotid artery (ICA) or proximal MCA occlusion within 6 to 24 hours since the last time they were seen well. The clinical core mismatch was defined based on the measurement of the ischemic core by CT along with a perfusion study or by the DWI sequence on MRI, stratified by age, as follows: ≥ 80 years of age – NIHSS ≥ 10 and core < 21 mL; < 80 years of age – NIHSS ≥ 10 and core < 31 mL; and < 80 years of age – NIHSS ≥ 20 and core between 31 mL and 50mL. 

 Thrombectomy was performed with a Trevo (Stryker, Kalamazoo, MI, US) device, without the possibility of using rescue therapy. The coprimary outcomes were the mean score for disability on the utility-weighted mRS, with values from 0 (death) to 10 (no symptom or disability), as well as an mRS score ranging from 0 to 2. ^45^ The median NIHSS score was 17 in both groups. The mean score on the utility-weighted mRS was 5.5 in the thrombectomy group and 3.4 in the control group. In addition, an mRS score ranging from 0 to 2 was achieved by 49% of patients undergoing the endovascular treatment, and by 13% of patients in the control group. Although there was greater clinical severity in this study, 47 the sICH and mortality rates were comparable between groups. 

**Figure 3:**
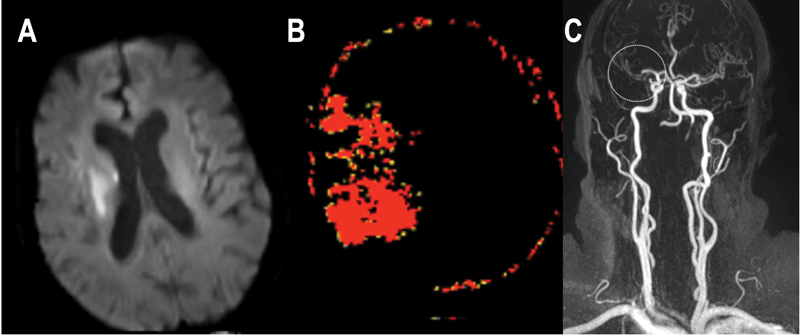
( **A** ) Diffusion-weighted imaging showing ischemic core < 70mL. The Tmax map by the perfusion study ( **B** ) shows mismatch volume > 15mL and the arterial angiographic study ( **C** ) demonstrates (dotted circle) obstruction in the right M1 segment.

 The DEFUSE-3 trial 48 evaluated the benefit of mechanical thrombectomy in the treatment of occlusion in the same arterial segments as those in the DAWN, but with a time window between 6 hours and 16 hours since the last time the patient was seen well. The imaging protocol included CT perfusion or MRI diffusion and perfusion. The definition used for core-penumbra mismatch required an ischemic core and a penumbra volume < 70 mL and > 15 mL respectively, in addition to a penumbra-to-core volume ratio > 1.8. Patients were randomized to receive mechanical thrombectomy plus standard medical care or standard medical care alone. The median NIHSS score was 16 in both groups. Patients undergoing EVT had better results in the distribution of functional outcomes in the mRS at 90 days as compared with clinical treatment alone (OR: 2.77; *p*  < 0.001). In addition, the percentage of patients with an mRS ranging from 0 to 2 was 45% in the EVT group versus 17% in the medical treatment group ( *p*  < 0.001). 48 Figure 3 demonstrates an example of core-penumbra mismatch that could be elegible for mechanical thrombectomy according to the DEFUSE-3 criteria. 

 The positive results obtained in the DEFUSE-3 48 and DAWN 47 trials motivated a new recommendation in the 2019 AHA/ASA guidelines 36 to perform CTA and CT perfusion, or magnetic resonance angiography (MRA) with DWI in association with MRI perfusion imaging or not for patients who are candidates for mechanical thrombectomy between 6 hours and 24 hours since the last time they were seen well (class I; level of evidence A). These should also be included in the EVT if the eligibility criteria in one of either study are met (class I; level of evidence A). 36

Table 1 summarizes phase-3 trials with proven efficacy for the use of intravenous thrombolysis and mechanical thrombectomy in unknown-onset stroke. 

**Table 1:** Summary of the positive phase-3 trials related to intravenous thrombolysis and mechanical thrombectomy in unknown-onset stroke

Study	Study treatment	Time window (hours)	Image paradigm	Clinical inclusion criteria	Image inclusion criteria	Randomized patients	Primary outcome at 90 days (intervention versus control)	NNT
WAKE-UP ^33a^	Alteplase	4.5	DWI-FLAIR mismatch	• 18–80 years of age; • measurable disabling neurological deficit	• Positive DWI and negative FLAIR	503	mRS 0–1: 53.3% versus 41.8% (OR: 1.61; 95%CI: 1.09–2.36; *p* = 0.02)	9
EXTEND ^43b^	Alteplase	4.5–9	Core and penumbra mismatch	• ≥ 18 years of age; • prestroke mRS < 2; • NIHSS score: 4–26	• Core volume < 70mL; • mismatch volume > 10mL; • mismatch ratio > 1.2	225	mRS 0–1: 35.4% versus 29.5% (OR: 1.44; 95%CI: 1.01–2.06; *p* = 0.04)	17
DAWN ^47c^	Thrombectomy	6–24	Clinical core mismatch	• ≥ 18 years of age; • stroke without response/contraindication to rt-PA; • prestroke mRS < 2; • NIHSS score ≥10; • Anticipated life expectancy of at least 6 months	• ICA or MCA-M1 occlusion; • core < 21mL and NIHSS score ≥ 10 (≥ 80 years old); • Core < 31mL and NIHSS score ≥ 10 (< 80 years old); • Core between 31 mL and 50 mL and NIHSS score ≥ 20 (< 80 years old)	206	Mean mRS score: 5.5 versus 3.4 (adjusted difference: 2.0; 95%CI: 1.1–3.0; posterior probability of superiority > 0.999)*	2.8
DEFUSE-3 ^48c^	Thrombectomy	6–16	Core and penumbra mismatch	• 18–85 years of age; • anterior circulation stroke; • prestroke mRS ≤ 2; • NIHSS score ≥ 6	• ICA or MCA-M1 occlusion; • core volume < 70mL; • mismatch volume ≥ 15mL; • mismatch ratio > 1.8	182	Median mRS score: 3 versus 4 (common OR: 2.77; 95%CI: 1.63–4.70; *p* < 0.001)	3.6

Abbreviations: 95%CI, 95% confidence interval; DAWN, Diffusion Weighted Imaging or Computerized Tomography Perfusion Assessment with Clinical Mismatch in the Triage of Wake Up and Late Presenting Strokes Undergoing Neurointervention; DWI, diffusion-weighted imaging; DEFUSE-3, Endovascular Therapy Following Imaging Evaluation for Ischemic Stroke 3; EXTEND, Extending the Time for Thrombolysis in Emergency Neurological Deficits; FLAIR, fluid-attenuated inversion recovery; ICA, internal carotid artery; M1, first segment of the middle cerebral artery; MCA, middle cerebral artery; mRS, modified Rankin scale; NIHSS, National Institutes of Health Stroke Scale; NNT, number needed to treat; OR, odds, ratio; rt-PA, recombinant tissue plasminogen activator; WAKE-UP, Efficacy and Safety of MRI-based Thrombolysis in Wake-up Stroke.

Notes: ^a^ Early discontinuation due to lack of funding; ^b^ Early discontinuation after WAKE-UP results; ^c^ Early discontinuation after interim analysis; *The DAWN trial used an adapted design based on a Bayesian linear model.

## FUTURE IMPLICATIONS FOR APPROACHING UNKNOWN-ONSET STROKE

 The extension of the treatment window for intravenous and endovascular treatments based on advanced neuroimaging concepts has gained new contours, especially after 2018, with the publication of the WAKE-UP 33 and EXTEND 43 trials for thrombolysis, and the DAWN 47 and DEFUSE-3 48 trials for mechanical thrombectomy. However, some limitations related to time and the method itself still need to be considered. 

 Concerning time, efficacy studies with an extended therapeutic window and defined upper time limit concluded that thrombolysis could be performed within 9 hours, and mechanical thrombectomy, within 24 hours. 43 47 Some recent studies 49 50 have concluded that thrombectomy appears to be safe if performed after 24 hours; still, due to their retrospective nature and small samples, this needs to be better validated. 

 Regarding the method used in the published studies, a central issue to be analyzed is its reproducibility. First, in order for the concepts of core-penumbra mismatch and clinical core mismatch to be applied, suitable software for manual or automated processing are required. In addition, there are limitations to the use of automated processing, such as overestimating the lesion volume in perfusion or processing erroneous results due to image quality, which ultimately affects the therapeutic decision. 51 Second, the WAKE-UP trial 33 has conditioned the treatment decision to performing only MRI scans, which, in most countries, has its availability limited to tertiary services. In turn, CT is more widely distributed and accessible, which makes it imperative to identify new markers to characterize the ischemia duration or potentially salvageable brain tissue by using this method. 

Third, understanding that the dynamic changes in neuroimaging studies reflect the pathophysiological process of the ischemic injury, which involves the stages of cytotoxic, ionic, and vasogenic edema, other alternative methods are currently being evaluated to estimate the time of stroke onset.

 By combining CT and CT perfusion, a German study 52 aimed to correlate the quantitative water uptake in the lesion area with stroke onset time within and after 4.5 hours. With the cut-off value of water uptake of 11.5%, the researchers were able to distinguish the ischemic time with a sensitivity of 98.6% and a specificity of 90.5%. 

 A subsequent study 53 compared the CT-based quantitative water uptake marker to the DWI-FLAIR mismatch as measured by MRI, and found an accuracy of 86%, sensitivity of 91%, and specificity of 78% with a previously-defined water uptake threshold of 11.5%, all of which were comparable to values obtained by MRI. 

 Relying on the DWI-FLAIR mismatch pattern as a guide for where to induce thrombolysis, in an Austrian retrospective study, 54 the researchers decided to include in the thrombolytic treatment group patients who were partially positive in FLAIR, as defined by the signal change in this sequence, but clearly less area than the corresponding DWI lesion and absent in the contralateral hemisphere. Sixty-four thrombolyzed patients with this imaging pattern were compared with a non-thrombolized control group by using clear positivity in FLAIR. Despite the methodological issues of this study, 54 the frequency of sICH and the functional outcome were comparable between the groups, opening the possibility for further studies that validate the partial positivity of FLAIR as a safe biomarker to perform thrombolysis. 

 In turn, to assess the visual and quantitative performance of MR-based methods as predictors of the time of onset of stroke, McGarry et al. 55 compared the signal intensity ratios of the T2-weighted sequences, T2 relaxation, DWI, apparent diffusion coefficient (ADC), and FLAIR, in addition to DWI-FLAIR mismatch. The study concluded that the T2 relaxation time was the most accurate measurement to estimate the time of onset of ischemia and that, taken together with the quantification of the ADC map to identify the lesion, it may be sufficient for patients with unknown-onset stroke, for it presents even better accuracy than does the DWI-FLAIR mismatch. 

 Finally, in addition to comparing new brain imaging modalities with those already validated, other studies have been seeking to expand the therapeutic arsenal against acute ischemic stroke. Due to its pharmacological properties and ease of administration, research on tenecteplase has emerged in recent years. 56 57 Three studies 58 59 60 related to unknown-onset stroke are ongoing. The Tenecteplase in Wake-up Ischaemic Stroke Trial (TWIST) 58 is a phase-3 study which aims to randomize 600 patients and assess the benefit of 0.25 mg/kg of tenecteplase within 4.5 hours of awakening with stroke symptoms, based on CT and CTA images (if possible). The Tenecteplase in Stroke Patients Between 4.5 and 24 Hours (TIMELESS) study, 59 which is also in phase 3, adopts theDEFUSE-3 imaging criteria 48 and is recruiting patients with stroke and LVO (ICA or MCA) to assess 0.25 mg/kg of tenecteplase within 4.5 hours and 24 hours since the onset of symptoms. Unlike the others, the Chinese Acute Tissue-Based Imaging Selection for Lysis In Stroke – Tenecteplase (CHABLIS-T) 60 is a phase-2 study comparing doses of 0.25 mg/kg and 0.32 mg/kg in stroke patients within 4.5 hours and 24 hours since the onset of symptoms as assessed by perfusion imaging. 

## PROPOSING A PROTOCOL TO APPROACH UNKNOWNONSET STROKE

 In the context of acute ischemic stroke, establishing an assessment protocol in the emergency room for patients suspected to have ischemia is mandatory (class I; level of evidence B). 36 Furthermore, as a clear time of symptom onset cannot be established in 20% of the cases, stroke teams must be able to investigate them promptly, to offer reperfusion therapies to the greatest number of patients. 

In summary, the studies that have shown the benefit of intravenous thrombolysis and mechanical thrombectomy in this population used perfusion CT or MRI with or without perfusion. Understanding that perfusion studies are dependent on manual or automated processing by software and that contrast injection is necessary, we have chosen, in the suggested protocol, to make the MRI the modality of choice to investigating unknown-onset stroke.

The strengths of the MRI are the possibility of establishing a protocol that is faster to execute and yields a quicker final response by including only the DWI, FLAIR, T2* (or another hemorrhage-sensitive) sequences and time-of-flight (TOF) angiography, which directly address the questions regarding whether a treatment is possible or not, as well as regarding the modality thereof that can be used. In the real world, probably the biggest downside is its unavailability in smaller centers.

 If the patient does not have contraindications to undergo an MRI scan, the criteria of the WAKE-UP trial ^33^ should be evaluated to consider eligibility for intravenous thrombolysis, or the criteria of the DAWN trial 47 when LVO is suspected. If it is impossible to perform MRI scans, either due to contraindication unavailability, perfusion CT is the next best option and helps in the inclusion of patients by the presence of a core-penumbra mismatch. 

The CT protocol starts with a scout view followed by non-contrast head CT, intracranial and cervical angiotomography (CTA), and then, perfusion CT. After non-contrast slices, a volume of 35 mL to 50 mL of iodinated contrast followed by a 20 mL saline chase is injected into a vein ideally at or above the antecubital region or the forearm to acquire the CTA. After that, another 30 mL to 50 mL of intravenous contrast is necessary to acquire perfusion images. Full brain coverage either by 2 perfusion slabs or by single perfusion (which is available in modern scanners), is mandatory.

 Automated perfusion processing software have been used to accelerate data availability and reduce interobserver variability. The RAPID CTP (iSchemaView, Menlo Park, CA, US) has been used and validated in the EXTEND, 43 DEFUSE-3, 48 and DAWN 47 trials. In Brazil, it was used in the RESILIENT trial. 61 However, its use requires evaluation of the vascular time-attenuation curves, generated from the selection of an arterial input function (AIF) and a venous output function (VOF). The selection of a large-caliber artery (carotid terminus, anterior cerebral artery, and proximal middle cerebral artery) and a large dural venous sinus (torcular Herophili, transverse sinus, or superior sagittal sinus) such as the AIF and VOF respectively, is recommended. 

Using deconvolutional analysis, postprocessing software platforms can provide measures of cerebral blood flow (CBF), cerebral blood volume (CBV), mean transit time (MTT), time-to-peak (TTP) and time-to-maximum (Tmax). The maps and thresholds used to estimate the ischemic core and penumbra are relative to CBF <30% of the contralateral hemisphere, and to Tmax > 6 seconds respectively.

**Figure 4:**
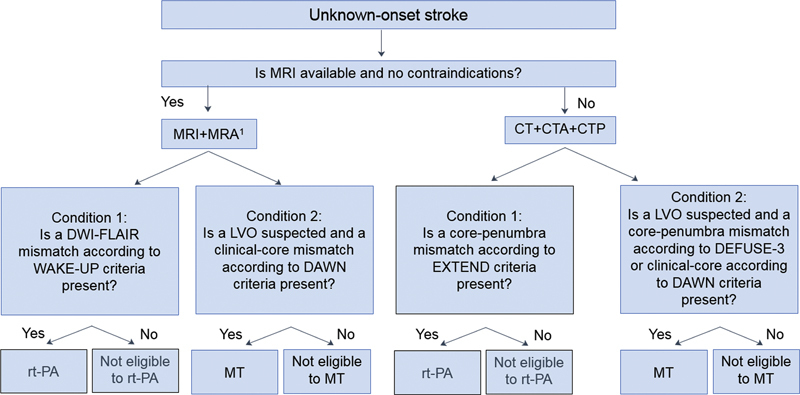
Flowchart for an advanced image-guided therapeutic approach to unknown-onset stroke. Abbreviatons: CT, computed tomography; CTA, computed tomography angiography; CTP, computed tomography perfusion; LVO, large vessel occlusion; MRA, magnetic resonance angiography; MRI, magnetic resonance imaging; MT, mechanical thrombectomy; rt-PA, recombinant tissue plasminogen activator. Notes: rt-PA – alteplase 0.9 mg/kg (maximum 90 mg); ^1^ Includes DWI, FLAIR, T2*, and TOF angiography.

 For perfusion CT, the same criteria as those used in the EXTEND trial 43 will be used to consider intravenous thrombolysis. In case of LVO, the DAWN 47 and DEFUSE-3 48 trials guide the selection of patients for mechanical thrombectomy. A flowchart to approach unknown-onset stroke is proposed in Figure 4 . 

 It is necessary to emphasize that, in patients with a time of symptom onset shorter than 4.5 hours, non-contrast CT is effective to exclude hemorrhage and, in the absence of other contraindications, to indicate the thrombolytic treatment. 36

 If an LVO within 6 hours since the last time the patient was seen well is suspected, the association of CT and CTA is sufficiently recommended to select the patient to undergo a mechanical thrombectomy. 36 Nevertheless, the RESILIENT trial, 61 which showed the benefit of mechanical thrombectomy plus standard care for patients within 8 hours of symptom onset, used CT or CTA in 99.1% of the intervention group, showing that this imaging modality may be suitable for this longer time window than otherwise stated in the guidelines. 

In conclusion, despite being a frequent condition in the clinical practice, unknown-onset stroke recently crossed the line of conservative clinical treatment to a level of multiple approaches depending on the time of symptom recognition, the availability of advanced imaging methods, and the expertise of the team, all of which are key to ensure that the best treatment is provided.

 Due to the publication of the four main positive trials on efficacy (WAKE-UP, 33 EXTEND, 43 DAWN, 47 and DEFUSE-3 48 ), intravenous thrombolysis can be offered to this population because of the evidence from DWI-FLAIR or core-penumbra mismatch, and mechanical thrombectomy due to the evidence from clinical core or core-penumbra mismatch. 

The need to identify new possibilities to extend the therapeutic window for patients with unknown-onset stroke is evident. This should be achieved with the identification of new neuroimaging modalities or the establishment of new criteria for previous positive studies, so that the treatment they propose becomes increasingly more available to everyone. In addition, depending on their own possibilities, national health systems need to structure themselves to expand the supply of human and technological resources for the approach to acute ischemic stroke and thus identify potential candidates for reperfusion therapies who, until recently, were excluded due to the strict time window they involved.
